# Review: Cross-breeding, advanced reproductive technologies, and genetic selection in twelve dairy production systems in Africa

**DOI:** 10.1016/j.animal.2025.101424

**Published:** 2025-03

**Authors:** E.K. Kathambi, T.S. Sonstegard, P.A. Larsen

**Affiliations:** a Department of Veterinary Biomedical Sciences, University of Minnesota, St. Paul, MN, 55108, USA; b Acceligen^TM^, 3388 Mike Collins Dr. Eagan, MN 55121, USA

**Keywords:** Artificial insemination, Dairy cattle, Embryo transfer, *In vitro* fertilization, Multiple ovulation

## Abstract

The supply and demand for milk in Africa have been rising due to factors such as population growth, urbanization, increasing incomes, and improved living standards. Dairy production significantly impacts the economic and social development of African countries, contributing over 10% to the agricultural gross domestic product in countries such as Ethiopia, Kenya, and Tanzania. The ongoing development of the African dairy industry can be attributed, to a certain extent, to the utilization of cross-breeding strategies and advanced reproductive technologies such as artificial insemination, embryo transfer, and the implementation of genomic selection programs. This review evaluates historical dairy breeding practices in twelve African countries and examines the use of advanced breeding tools to enhance and sustain cattle breeding programs across Africa. Eighteen cases from twelve African countries are reviewed to highlight the successes and reported positive impacts on dairy production, revenue, livelihoods, and genetic diversity. We have pinpointed the challenges of integrating new breeding approaches and technologies for African dairy programs, such as insufficient funding, low return on investment in biotechnology, poor monitoring and evaluation of breeding programs, and lack of developed legal frameworks on biotechnological operations. We have recommended strategies to overcome these barriers that may be employed in dairy systems to improve the adoption of breeding technologies across the region.

## Highlights

• The review covers four dairy breeding techniques across 12 African countries.
• Successful dairy breeding programs can be adopted in other developing countries.
• Challenges in adopting breeding techniques can inform future breeding strategies.
• Limited funds and breeding data are key barriers to implementing breeding programs.
• Identified gaps offer opportunities for investment and innovation in Africa.

## Implications

Cattle breeding technologies successfully integrated across Africa, including cross-breeding with high-performance breeds, artificial insemination, embryo transfer, and genetic selection, can be adapted to other dairy production systems in the tropics. The challenges reported in the adoption of breeding technologies can be used to refine future dairy cattle breeding strategies in Africa and globally. Gaps identified in the review such as limited funding, poor collaboration, and limited follow-up in dairy breeding programs, provide opportunities for further engagement of other stakeholders in improving dairy production in Africa. The positive impact of breeding technologies on the dairy and economic development of Africa can be leveraged for additional investment and research opportunities in the region, through public and private partnerships.

## Introduction

Agriculture is a key sector in the twelve African countries reviewed herein, with contributions to their gross domestic product (**GDP**) (Table 1) ranging from 1.8% in Botswana to 37.6% in Ethiopia (World Bank, 2023a). Between 2010 and 2020, 43–49% of Africa’s workforce was employed in agriculture (OECD, 2021), with over 50% of agricultural workers being women in countries like Rwanda, Uganda, and Kenya (Faostat, 2022). Dairy production is particularly important, contributing over 10% to agricultural GDP in countries such as Kenya, Tanzania, and Ethiopia (MoITI, 2023, Yilma and Mounde, 2023, KDB, 2024, Land O’ Lakes Venture 37, 2024). In 2021, the gross production value of raw cattle milk in Africa was estimated at over USD 14 billion (Faostat, 2023a).

**Table 1:** Contribution of agriculture to the Gross Domestic Product (GDP) in twelve African countries.

Country	Contribution of agriculture to national GDP in 2021
Burkina Faso	18.5%
Botswana	1.8%
Cameroon	17%
Ethiopia	37.6%
Kenya	21.2%
Malawi	22.7%
Rwanda	24.1%
Senegal	15.3%
South Africa	2.6%
Tanzania	25.9%
The Gambia	22.4%
Uganda	23.8%

Data source: World Bank, 2023b.

Africa is home to dynamic and diverse dairy farming systems comprising of smallholder dairy systems (1–10 animals) that make up 70% of the dairy industry in most countries, medium-scale systems (11–50) that can be involved in intensive or semi-intensive production, and large-production systems (> 50) (Peeler and Omore, 1997, Staal et al., 1997, Omore et al., 1999, Devendra, 2001, Ibeagha-Awemu et al., 2019). These dairy systems are practiced under varying environmental conditions with different cattle breeds performing differently in these environments.

The demand and supply of milk in Africa have grown due to population increases, urbanization, rising incomes, and lifestyle changes (Thornton, 2010, AUIBAR, 2016). Milk production rose from 23 × 10^6^ tons in 2001 to 42 × 10^6^ tons by 2021 (Faostat, 2023b). The milk production across the 12 countries in the review is summarized in Table 2. The per capita milk consumption, currently around 30 L annually, is expected to increase to 64 L by 2050 (AUIBAR, 2016). The growth of Africa's dairy industry is partly due to cross-breeding programs and advanced reproductive technologies (**ARTs**) such as artificial insemination (**AI**), embryo transfer (**ET**), and genomic selection. In Kenya, AI in dairy cattle resulted in an 11% monetary gain, while the use of ET or *in vitro* fertilization (**IVF**) led to an 184% increase in economic returns (Gicheha et al., 2019). Cross-breeding involves the production of a hybrid animal by mating individuals from different breeds, lines, or populations (Sørensen et al., 2008). The sustainability of cross-breeding programs is dependent on assured access to adequate breeding stock, the opportunity for the hybrids to express their genetic potential, and the availability of a reliable marketing chain where the hybrids can be integrated (Leroy et al., 2016).

**Table 2:** Raw cattle milk produced in twelve African countries between 2000 and 2022.

	Annual cattle raw milk yield (tonnes)							
Country	2000	2005	2010	2015	2019	2020	2021	2022
Burkina Faso	471 340	415 973	472 111	339 479	200 192	177 571	215 685	211 823
Botswana	74 192	117 621	129 861	200 000	220 000	216 287	220 716	225 235
Cameroon	155 677	161 390	175 000	200 000	208 000	188 000	220 666	205 555
Ethiopia	900 000	2 138 980	4 057 998	3 200 282	3 895 323	4 692 994	3 866 052	4 151 456
Kenya	2 224 000	3 752 200	3 638 592	3 444 214	3 983 250	4 048 116	4 640 860	4 214 874
Malawi	35 000	42 541	46 672	52 113	63 058	64 876	66 893	67 791
Rwanda	107 905	120 000	172 345	174 351	179 105	198 580	187 795	181 400
Senegal	100 601	100 170	155 822	210 000	225 000	220 666	222 222	222 629
South Africa	2 306 000	3 044 000	3 123 000	3 538 435	3 873 000	3 853 000	3 825 000	3 771 000
Tanzania	710 000	1 386 400	1 649 857	2 058 726	2 278 461	3 002 555	3 101 384	3 448 072
The Gambia	60 325	68 157	70 303	78 851	52 335	49 086	48 882	48 678
Uganda	685 956	1 317 804	141 800	1 616 000	1 683 314	1 660 392	1 671 294	1 674 018

Data source: FAOSTAT, 2024.

Broadly, ARTs refer to the various biotechnologies developed to obtain multiple offspring from genetically superior animals. These include techniques such as AI, multiple ovulation (**MO**), ET, IVF, somatic nuclear transfer, and cloning. In this review, ARTs consist of AI, MOET, and IVF. These techniques promote rapid genetic progress and reduce disease transmission risks compared to natural mating (Rodriguez-Martinez, 2012). Advances like AI with sexed semen allow producers to select desired offspring, though this technology has limitations, such as reduced fertility rates in sexed semen (Hernandez Gifford and Gifford, 2013).

Multiple ovulation, also referred to as superovulation, is the hormonal manipulation of donor females to release multiple eggs during a single estrus cycle using hormones such as Gonadotropin-releasing hormone, follicle-stimulating hormone and prostaglandin F2α (Phillips and Jahnke, 2016). Embryo transfer involves collecting an embryo from a donor female and implanting it into a recipient female for development. (Stroud, 2012). Ideally, ET can accelerate genetic improvement in cattle within 5 years (Rodriguez-Martinez, 2012). By combining MO and ET, farmers can potentially obtain up to 32 embryos per cow annually, a significant improvement compared to traditional breeding, where only one calf is produced yearly, with uncertain sex outcomes (Steel and Hasler, 2003, Glenn, 2004).

*In vitro* fertilization in cattle involves the recovery of eggs from superior females and fertilizing them with spermatozoa from superior sires outside the body. The resulting embryos are implanted into recipient surrogate dams (Hernandez Gifford and Gifford, 2013). Despite its potential, the adoption of IVF technology is low in many regions due to high costs, the need for specialized infrastructure, and the advanced skills required for its successful use in cattle breeding programs (Hernandez Gifford and Gifford, 2013).

Genomic selection uses genomic estimated breeding values, calculated from single nucleotide polymorphism (**SNP**) effects derived from predictive equations based on a reference population with known SNP genotypes and traits (Schefers and Weigel, 2012). Before genomic testing, breeding decisions were based on progeny testing, a lengthy and expensive process that involved tracking the performance of offspring over time (Schefers and Weigel, 2012). Bovine SNP assays, developed over the years, provide faster evaluations with reliabilities ranging from 60–70% (Schefers and Weigel, 2012). High-density assays like BovineSNP50 analyze over 50 000 markers with high accuracy (Matukumalli et al., 2009), while lower-density assays like the Bovine LD chip offer cost-effective options for smaller-scale farming systems (Boichard et al., 2012). The choice of assay depends on the specific farming goals, scale, and economic considerations.

**Fig. 1:**
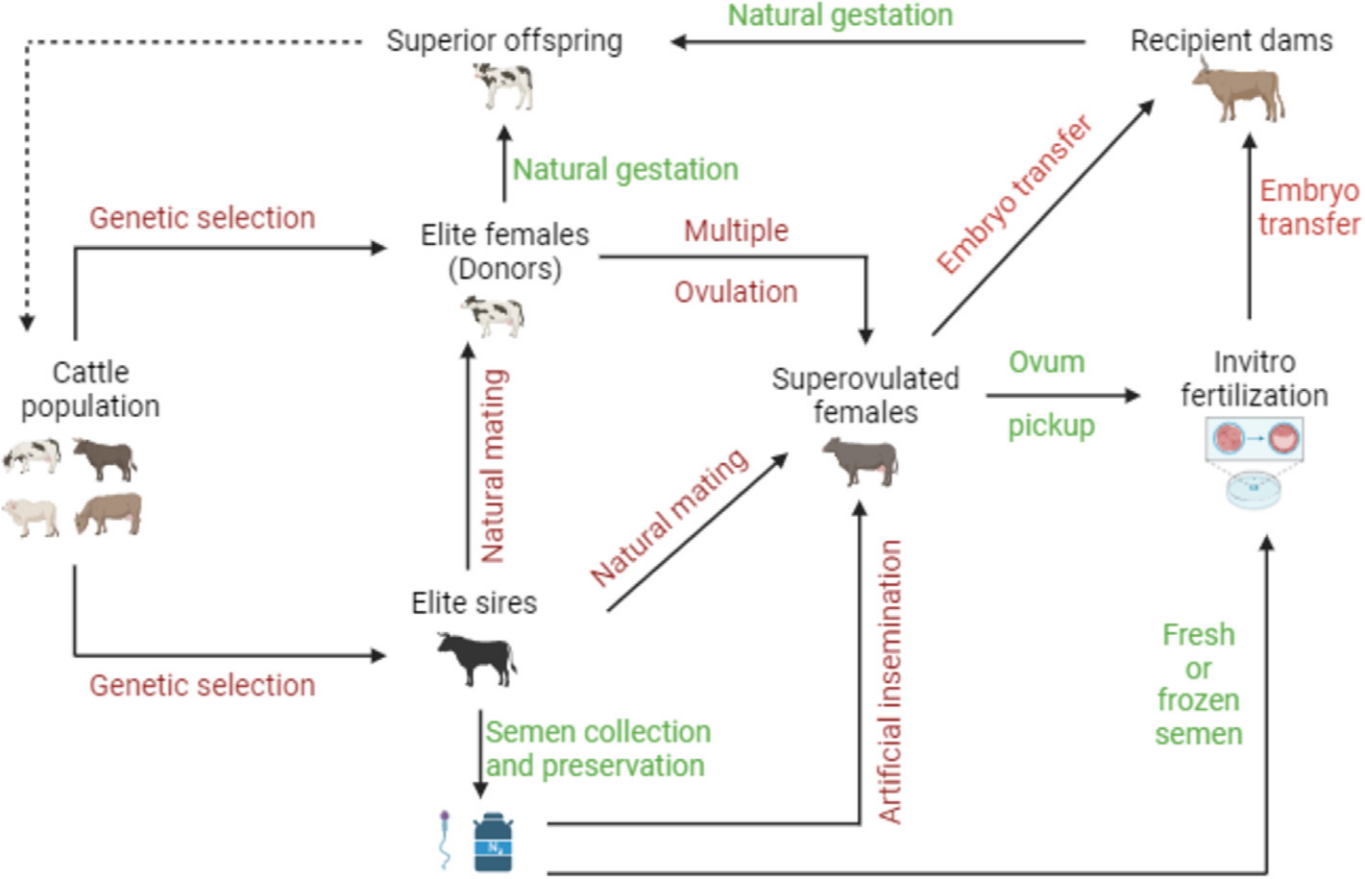
Synergistic application of advanced reproductive Techniques and genetic selection in global cattle production systems; Created with Biorender.com.

The successful integration of cross-breeding, advanced reproductive technologies, and genomic marker selection into African cattle dairy programs has positively impacted production, revenue, livelihoods, and genetic diversity (Marshall et al., 2019). These technologies work synergistically to enhance productivity, promote genetic progress, and shorten generation intervals (Fig. 1). This review aims to assess and collate the available information on the reported use of cross-breeding, ARTs, and genetic selection in African dairy breeding programs. We provide case studies highlighting their successful application in improving cattle productivity and developing the African dairy industry.

## Literature search methodology

**Fig. 2:**
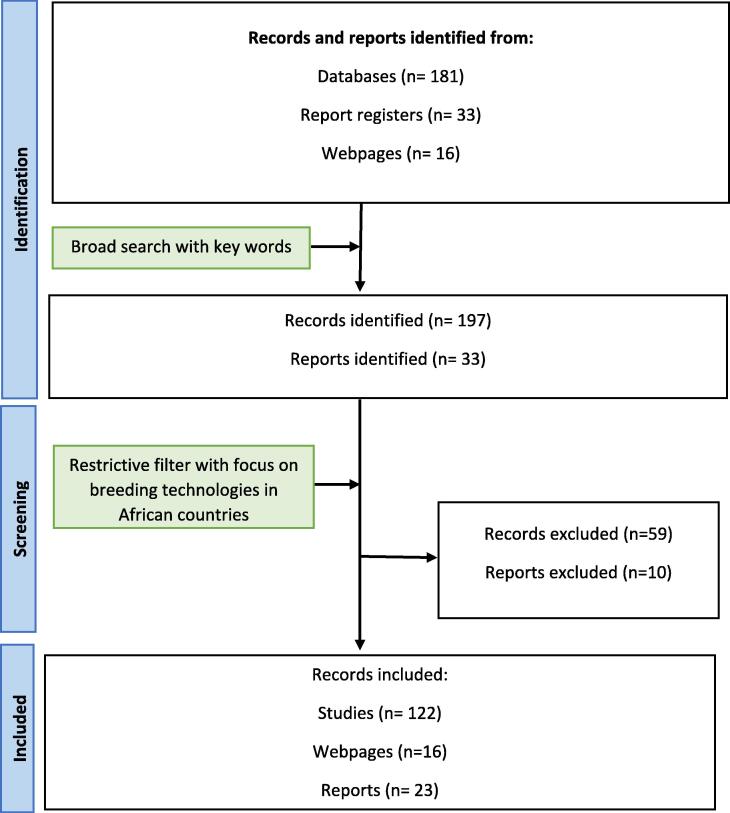
Modified PRISMA chart illustrating the selection of literature included in the review of reproductive technologies in African dairy production systems.

**Fig. 3:**
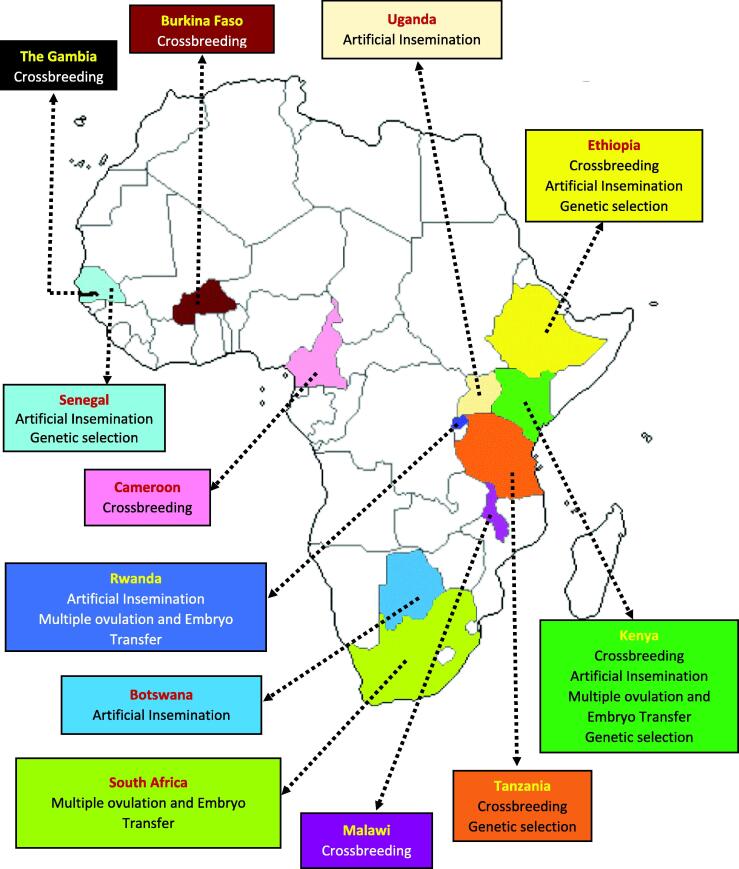
Geographic distribution of the African dairy production case studies reviewed herein.

Peer-reviewed journal articles, conference papers, reports, and book chapters used in this review were sourced from public online databases including Google Scholar®, Pubmed®, Elsevier®, and FAOSTAT®. Additional print resources were acquired from organizational websites, news articles, and other relevant sources. Photos and graphics used in this document are cited based on their source. A broad search using the keywords “Livestock sector”, “Genetic improvement of cattle”, “Breeding technologies”, “Adoption of cattle breeding technologies”, “Impact of cattle breeding technologies”, “Challenges/constraints in the use of breeding technologies”, “Biotechnology”, “Cross-breeding of cattle”, “Artificial insemination of cattle”, “Embryo transfer of cattle”, “Genetic selection of cattle” was performed. These searches were performed multiple times together with the name Africa and the names of African countries. All searches were performed from July 2023 to January 2024. More restrictive inclusion criteria were also used to narrow down the resources where the articles with the above keywords and specific to Africa or African countries were considered (Fig. 2). We note that limited published data on animal populations and quantifiable impacts of breeding technologies were available for central and northern African countries, thus preventing inclusion herein. A total of nineteen cases are discussed from twelve African countries (Fig. 3).

## Impact of using breeding technologies in African dairy systems

### Cross-breeding

In developing countries, cross-breeding initiatives began in the late 19th to early 20th centuries to improve indigenous breeds and create new ones (Madalena et al., 2002). When adapted to local environmental conditions, cross-breeding has improved animal performance and farmer incomes (Roschinsky et al., 2015). The current African taurine and zebu (indicine) genotypes have been influenced by zebu migrations from Asia and Arabia over the years (MacHugh et al., 1997). It is stipulated that the humpless taurine Longhorns (*Bos taurus*) were the first cattle to be introduced in Africa around 6000 BC. Zebu introgression into African cattle populations occurred mainly through male-mediated genetic exchange, where zebu bulls mated with taurine females (MacHugh et al., 1997). Currently, Africa is home to about 180 recognized cattle breeds (Mwai et al., 2015) with about 146 of these being indigenous (Rege and Tawah, 1999).

Dairy cross-breeding programs in Africa leverage the desirable traits of *Bos indicus* (African humped cattle), known for their climatic adaptation, disease resilience, and longevity, alongside the superior productivity and growth traits of *Bos taurus* (African humpless cattle) and exotic breeds from regions like North America and Europe (Cunningham and Syrstad, 1987, Tawah et al., 1999, Karugia et al., 2001). Studies indicate that the success of cross-bred cows is not only solely due to their genetic makeup but is also heavily influenced by environmental factors and management practices (Rege, 1998, Ayalew et al., 2003, Chagunda et al., 2004, Mbisha and Kabululu, 2023). The improved production of crossbreeds must be weighed against the economic benefits for farmers to ensure the sustainability of biotechnologies in resource-limited, demand-driven systems. Several African countries have reported the integration of cross-breeding programs in cattle production systems with varying levels of success.

#### Burkina Faso

In partnership with various stakeholders, the national government initiated at least three cattle cross-breeding programs in the country, aimed at improving productivity and disease resilience of local breeds. The Fulani Sudanese Zebu and Pure Azawak Zebu cross-breeding project was implemented between 2000 and 2015, with support from the Belgian Technical Cooperation. Conducted across 11 sites with 300 farmers and 2400 animals, the program led to the formation of Azawak Zebu breed associations. The resulting milk yields were 625 kg for pure Azawak Zebu, 516 kg for F1 crosses, and 560 kg for pure Fulani Zebu (Ouédraogo et al., 2021).

In 2016, community-based breeding programs were launched using a farmer-integrated approach under the “Local cattle breeds of Burkina Faso-Characterization and use” project. These programs aimed to preserve and improve local cattle breeds, particularly the Baoule (Lobi) breed valued by communities for meat and trypanotolerance. Initially involving 100 farmers and 2 000 animals, young Baoule bulls were evaluated based on age-corrected BW and growth, with faster-growing bulls considered more trypanotolerant. In 2020, 70 bulls were selected for breeding from 200 candidates following three rounds of selection (Ouédraogo et al., 2021).

Initiated in 2016 by Ceva, the Vache du Faso project aimed to enhance milk production by cross-breeding local zebu cows with French Montbeliarde and Tarentaise breeds (Ceva, 2023). These sire breeds were chosen for their adaptability, thermotolerance, and efficient feed use. Between 2016 and 2019, 5 479 Zebu cows were inseminated with semen from 16 bulls, and 1 655 cross-bred calves were born and survived for at least 1 month (Ceva, 2023). A genetic strategy was later implemented to balance local and exotic genetics in future generations but faced challenges due to poor success with embryo implantation and limited demand for artificial insemination (Ceva, 2023, Allan et al., 2024).

#### Cameroon

The first cross-breeding of dairy cattle in Cameroon began in the 1930 s with German Brown cattle at Buea Upper Farms (Atekwana and Maximuangu, 1981, Tambi, 1991). In the 1970 s, Holstein-Friesian and Pinzgauer cattle from Austria replaced them, and Montbeliarde semen from France was used for cross-breeding with local cattle in Dschang and Jakiri (Tambi, 1991, Bayemi et al., 2005). A dairy research station was established in Bambui in 1964 to improve cattle resilience to extreme environmental conditions (Njwe, 1984, Tambi, 1991). In 1967, Brown Swiss heifers were cross-bred with N’Dama cattle, and in 1975, Holstein-Friesian and Montbeliard semen was imported for cross-breeding with Gudali cattle. By 2005, Heifer International was importing Jersey and Holstein cattle for further improvements (Bayemi et al., 2005). Considering calving at first insemination and calving interval, Holstein-Gudali crossbreeds showed the best reproductive performance (Flora et al., 2019).

#### Ethiopia

In 1968, a cross-breeding program at Asela station in Ethiopia produced F1 hybrid heifers with 50% local breeds (*Bos indicus*) and 50% exotic breeds (*Bos taurus*). Indigenous cows (Fogera, Barca, and Boran) sourced locally were crossed with Friesian and Jersey breeds imported from Kenya. Over time, cross-breeds were upgraded to 50, 75, and 87.5% Friesian, with Jersey-crosses being less favored due to milking difficulties. On evaluation, cross-breeds outperformed indigenous breeds in milk yield, reproductive performance, milk quality, and maintenance estimate (Kiwuwa et al., 1983). Several projects followed, including the Selale Peasant Dairy Development Pilot Project initiated in 1987 and scaled up in 1995 as the Small-holder Dairy Development Project. Between 1995 and 1999, the project distributed 167 cross-bred heifers and 58 breeding bulls to small-holder farmers. Additionally, 4 859 cows were bred with improved bulls producing 1 093 calves (Desta, 2002). In 2007, the Improving Productivity and Market Success project began cross-breeding local Zebu cows with Borana bulls in Metema district (Tegegne et al., 2016). The first Borana cross-bred calves were born in 2008, followed by mass estrus synchronization and insemination efforts. A field day in 2013 showcased the performance of the Borana cross-breeds (Tegegne et al., 2016).

#### The Gambia

A pure-breeding program for N’Dama cattle in Gambia began in 1994, led by the International Trypanotolerance Centre with support from Germany (Dempfle and Jaitner, 2000). Approximately, 1 200 N’Dama cattle were selected to form a nucleus breeding unit at International Trypanotolerance Centre sites in Kenaba and Bansang (Dempfle and Jaitner, 2000). The program involved the continuous addition of male offspring from superior cows screened for high milk yield, with 500–1 000 cows screened annually (Dempfle and Jaitner, 2000). By 2000, the program aimed to produce young bulls with improved production traits compared to local bulls (Dempfle and Jaitner, 2000). In parallel, a cross-breeding program initiated in 1994 sought to enhance N’Dama cattle using Jersey and Holstein-Friesian breeds. In 1999, 187 N’Dama cows were inseminated with European semen, resulting in 40 cross-bred calves born in 2000. By 2001, nine F1 cows were distributed to nearby peri-urban farmers. F1 cows produced up to five times more milk than N’Dama cows, with Holstein-Friesian crosses yielding more than Jersey crosses. With proper management, F1 cattle thrived in Gambia's local environmental conditions (Diack et al., 2005).

#### Kenya

Cross-breeding indigenous Zebu cattle with the Sahiwal breed in Kenya began in 1939 to develop a dual-purpose breed that could be utilized for milk and meat in the country. The initial breeding stock was established at 13 livestock improvement centers, comprising Sahiwal bulls and cows imported from Pakistan and India, along with local Zebu cows. Additional Sahiwal bulls were imported in 1945 and 1964, and the cross-bred Sahiwal cattle (Kenya Sahiwal) were systematically bred with pure Sahiwal bulls. By 1962, there were 2 500 Sahiwals at the livestock improvement centers (Muhuyi et al., 1999). In 1963, the National Sahiwal Stud was founded at the National Animal Husbandry Research Centre in Naivasha. In 1991, 1 000 doses of semen from proven Sahiwal bulls were imported from Pakistan for use in the Sahiwal stud and other herds in the country (Muhuyi et al., 1999). The Kenya Sahiwal, now over 3 000 registered animals, is raised on ranches for meat and milk, with an average mature weight of 425 kg for cows and 500 kg for bulls. The breed's mean lactation yield is 1 574 kg, ranging from 972 to 2 940 kg (Muhuyi et al., 1999). An economic analysis of performance data from 1976 to 1996 on four crosses (Ayrshire, Brown Swiss, Friesian, and Sahiwal) in a Kilifi dairy ranch showed that the first crosses of Friesian and Sahiwal had the highest profit per day of productive life. In systems achieving 3 000 kg lactation yields, these crosses also excelled in traits like calving frequency. The study suggested that synthetic breeds based on Ayrshire, Brown Swiss, or Friesian could benefit smallholder dairy systems (Kahi et al., 2000).

#### Malawi

In the 1980 s, a program in southern Malawi aimed to develop 50% Friesian heifers using exotic Friesian bulls and various crossbred cows, including Sussex, Brahman, and Africander. At 6 months, the 50% of Friesian calves were moved from Chizombe to Chikowa and then to Tuchila at 1 year of age for insemination with imported Friesian semen. After calving, the cows were sold to smallholder farmers, while 75% of Friesian female calves were reared at Tuchila and inseminated at 2.5 years to produce 7/8 Friesian calves. This process continued for higher-grade Friesians, which were either retained for milking or sold. Performance evaluation showed that 75% of Friesian cows produced more milk than 50% of Friesian cows, although the latter calved 3 months earlier (Agyemang and Nkhonjera, 1986).

The Malawi Canada Dairy Development Project, initiated in 1979 by the government and the Canadian International Development Agency, imported 400 Canadian Holstein-Friesian cattle to crossbreed with local zebu (Chagunda, 2002). By 1997, over 6 000 Holstein-Freisian x Malawi Zebu cows were owned by smallholder farmers (Malawi Government, 1997, Chagunda, 2002). The overall impact of these cross-breeding programs was lower than expected, with simulations showing higher efficiency for Malawi zebu cows in low-input, low-output systems (Chagunda, 2002).

#### Tanzania

Since 1905, Tanzania has attempted to improve the Shorthorn Zebu for milk and meat. A structured breeding program began in 1935 using Ayrshires and other *Bos taurus* bulls, but the results were unsatisfactory. The program was modified to include Red Sindhi and Sahiwal breeds from India and Pakistan (Wilson, 2018, Wilson, 2021). By 1958, after many crossings, the Mpwapwa breed was established with a composition of 35% Red Sindhi, 20% Sahiwal, 20% Tanzania Shorthorn Zebu, 10% Boran, 5% Ankole, and 10% Ayrshire (Getz et al., 1986), aiming for 2 300 kg of milk in 305 days and 230 kg of steer carcass in under 4 years (Wilson, 2021).

By 1963, the offspring showed variability in traits and could not be distinctly classified as a breed (Wilson, 2018, Wilson, 2021). Between 1968 and 1971, Mpwapwa females were crossed with Friesian, Ayrshire, and Jersey bulls (Mkonyi, 1982), but the original goals were not fully achieved. The Mpwapwa population declined to fewer than 1 000, leading to an endangered classification by the FAO (Scherf, 2000), although some were introduced to smallholder farms (Komwihangilo et al., 2010, Komwihangilo et al., 2013). Genetic evaluations indicated a decline in progress at the Mpwapwa research station (Chawala et al., 2017). A recent study found that Mpwapwa calves had the lowest mean birth weight (22.6 kg) compared to four of its cross-breeds (Vianze (26.6 kg), Kikombo (25.7 kg), Matondwe (24.5 kg), and Chimbamba (24.4 kg) (Mbisha and Kabululu, 2023).

### Artificial insemination

Artificial insemination was first introduced in Kenya in 1935, and it was the first time the technology was introduced in Africa (Kouamo et al., 2009a). In other African countries, the first recorded insemination of cows was reported in the early 1960 s and has steadily gained traction in the continent since then. In 1998, using data from 29 countries, AI resources across the African region were distributed as 18 collection centers, 161 semen banks, and 646 bulls. An estimated 55 × 10^6^ fresh doses and 1.4 × 10^6^ frozen doses were produced and processed in the region during this period (Thibier and Wagner, 2002).

Out of 42 countries in Africa, 74% of them reported using AI in 2007 while all Eastern and Southern African countries reported use of AI in 2015 (Rischkowsky and Pilling, 2007, Scherf and Pilling, 2015). Farmers in Africa have reported advantages of using AI over natural mating including, but not limited to, accelerated genetic turnover and improvement, lower cost relative to bull mating, forgone cost of rearing bulls, and decreased risk of disease transmission (Bulale, 2000, Baltenweck et al., 2004).

#### Botswana

Before 1960, Botswana faced cattle trade restrictions from South Africa, causing an economic crisis that ranked the country as the second poorest globally (Magang, 2015). The introduction of AI technology, alongside a bull subsidy scheme in 1964, helped grow the national cattle herd from 1.6 million in 1966 to 3 million by 1980 (Fidzani, 1985). To meet the rising AI demand, two AI stations were established in 1967, followed by the Ramatlabama AI bull station in 1970 with Danish funding. By 2011, 15 AI centers served about 7 283 cows annually (Moreki and Ranko, 2011). In 1994, an On-farm AI scheme was introduced to address transportation challenges, further boosting the cattle industry. Between 2011 and 2018, over 10 000 semen doses were distributed annually, maintaining stable conception rates (Moreki et al., 2019). Artificial insemination has significantly contributed to Botswana’s cattle industry, a major revenue source (Moreki and Ranko, 2011).

#### Ethiopia

The Chilalo Agricultural Development Unit began Ethiopia's dairy cattle genetic improvement in 1960 through AI, with the first frozen bovine semen produced in 1973. In 1981, the National Artificial Insemination Centre was established, importing semen from Holstein, Brahman, Friesian, and Jersey breeds (Yilma et al., 2011). Between 1984 and 2000, a total of 351 032 inseminations resulted in 120 684 calves (Shiferaw et al., 2002). Artificial insemination service use and semen production increased since 2001, despite a 2005 drop (Yilma et al., 2011), with average conception rates across regions ranging from 27–61% (Desalegn et al., 2009, Woldu et al., 2011, Ali et al., 2013, Ashebir et al., 2016, Melesse et al., 2020). In 2007, the Improving Productivity and Market Success project introduced hormonal estrus synchronization and mass insemination in Tigray, boosting pregnancy rates from 27–62%, and AI service efficiency from 6 to 45 inseminations per AI technician per week (Tegegne et al., 2016).

#### Rwanda

**Fig. 4:**
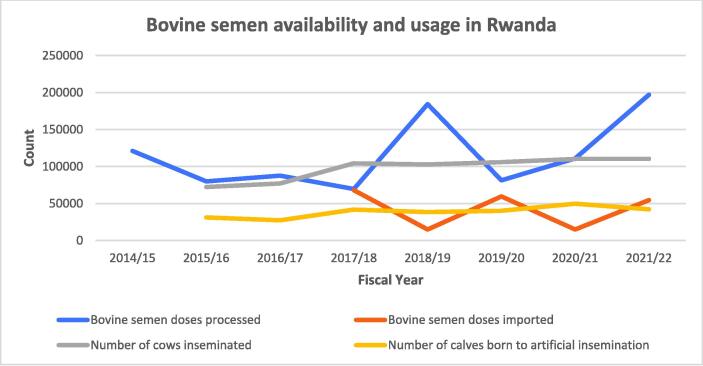
Bovine semen availability and usage in Rwanda, 2014–2022. Data sources: MINAGRI, 2015, MINAGRI, 2016, MINAGRI, 2017, MINAGRI, 2018, MINAGRI, 2019, MINAGRI, 2020, MINAGRI, 2021, MINAGRI, 2022.

Dairy genetic improvement in Rwanda is led by the National Artificial Insemination Centre, supported by partners like cattle farmers, NGOs, and livestock organizations. The Rwanda Agriculture and Animal Resources Development Board manages activities, including maintaining two liquid nitrogen plants (Ngamije, 2022). In 2017/2018, 69 715 semen doses were produced and processed domestically while 68 101 doses were imported under the Jersey Inka Nziza project (MINAGRI, 2018). Since then, semen produced in-country increased to 197 049 doses in the 2021/2022 fiscal year and the imported semen declined to 15 000 doses in the 2020/2021 fiscal year (MINAGRI, 2021, MINAGRI, 2022). Cows inseminated increased from 72 386 cows in 2014/2015 to 110 495 cows in 2021/2022, with calves born rising from 31 351 to 42 195 (MINAGRI, 2015, MINAGRI, 2022). However, the COVID-19 pandemic (2019–2021) caused a decline in semen production and AI success (Fig. 4). In 2017, Nyagatare district saw a 16.9% AI adoption rate, influenced by the proximity of farmers to AI centers (Mushonga et al., 2017).

#### Kenya

Dairy cattle improvement using AI in Kenya began during the colonial era with the first program launched in 1935 to control breeding diseases, inseminating about 6 000 cows in 2 years (Muriuki, 1993). The Central Artificial Insemination Station was established in 1946 to produce and distribute disease-free semen, increasing inseminated cows to 15 000 by 1947 (Mwangi, 1991, Muriuki, 1993). The 1954 Swynnerton Plan allowed commercial dairying, and AI services expanded to Central and Eastern provinces in 1956 (Muriuki, 1993, Thorpe et al., 2000). By independence in 1963, Kenya had approximately 4 × 10^5^ dairy cattle, including exotic breeds and their crosses with the East African Zebu (Thorpe et al., 2000). The Kenya National Insemination Service was founded in 1966, offering heavily subsidized AI services and introducing a roadside crush scheme for smallholder farmers (Muriuki, 1993). However, subsidies ended in 1993, leading to the privatization of AI services (EADD, 2013). This allowed dairy co-operatives to provide affordable AI services on credit deducted from milk income, preferred by farmers over private providers (Owango et al., 1998, Omondi et al., 2017).

**Fig. 5:**
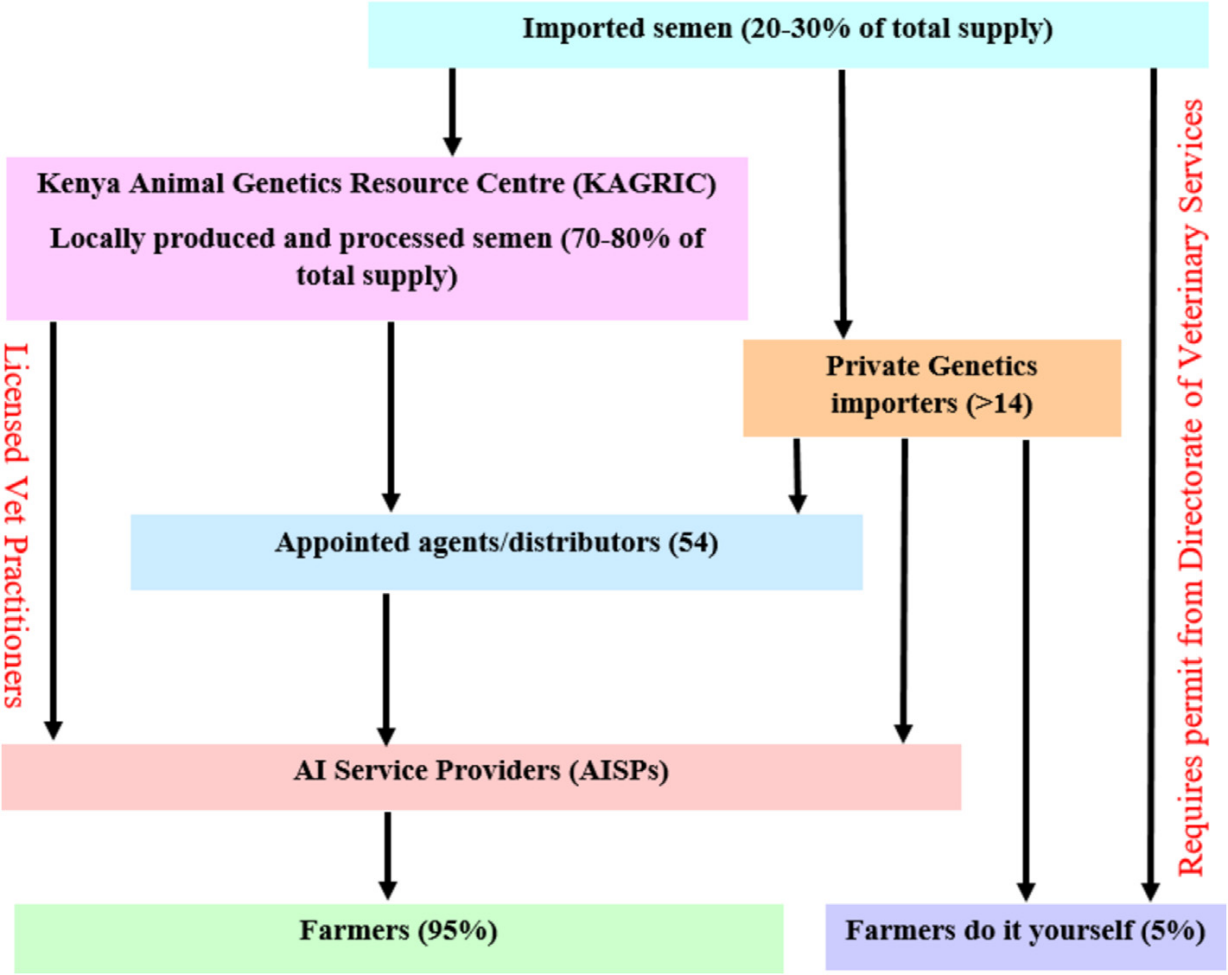
Bovine semen production, distribution, and delivery to artificial insemination service providers (AISPs) and farmers in Kenya. .

Source: Makoni et al., 2015

From 2006 to 2013, Kenya reported 1.7 million inseminations, with around 83% delivered by private AI service providers (Fig. 5). Approximately, 70% of inseminations went unreported (Makoni et al., 2015). Artificial insemination usage in 2014 ranged from 18–27% of total cattle breedings. The Kenya Animal Genetic Resource Centre reported production of 700 000–750 000 semen units from 125 bulls (Friesian, Ayrshire, Guernsey, and Jersey) in 2018/2019 and 2020/2021 (OAGK, 2019, OAGK, 2021), with a projected demand of 2 million units by 2023 (Makoni et al., 2015). A recent study showed a 13.3% adoption rate of AI among smallholder farmers in dryland areas, influenced positively by proximity to AI centers and access to information. However, concerns such as unprofessionalism among service providers hindered wider adoption (Abot, 2020).

#### Senegal

In 1964, the Senegalese government introduced the genetic improvement of Fulani Zebu (Zebu Gobra) through artificial insemination at the Centre for Zoo-technical Research in Dahra-Djoloff (Seck et al., 2016). By the 1990s, government and private operators delivered AI services and imported semen. Currently, semen and exotic breeds are distributed entirely by private operators (Seck et al., 2016). The National Centre for Genetic Improvement, established in 2005, produces semen using Normand, Holstein, and Montbeliarde bulls imported from France, along with additional germplasm from France and Brazil. Other available genotypes for cross-breeding include Limousine, Guzera, Gir, Nelor, Jersey, Charolaise, Brune des Alpes, Blonde d'Aquitaine, Girolando, and Abondance (Seck et al., 2016).

The livestock support project, initiated in 1992, aimed to boost milk and meat productivity in Senegal, focusing on AI for genetic improvement. Between 1995 and 2005, approximately 5 000 cows were inseminated, yielding pregnancy rates of 38–74% (Kouamo et al., 2009b). During this period, 380 calvings occurred from 1 373 inseminations. Following the Special Program for Artificial Insemination, the government aimed to inseminate 5 × 10^5^ cows by 2012, with an estimated 20% (1 × 10^5^ cows) successfully inseminated (Seck et al., 2016).

#### Uganda

Dairy cattle improvement in Uganda through AI began in the 1960 s, initially provided mainly by the government (Nakimbugwe et al., 2004, Eklundh, 2013). Today, AI services are offered by the National Animal Genetic Resources Centre and private partners, including NGOs (Eklundh, 2013). Artificial insemination usage increased from 5% in 2004 to 7% in 2008 but remains low compared to neighboring countries like Kenya (Eklundh, 2013). In the fiscal year 2021/2022, National Animal Genetic Resources Centre produced 135 584 semen units and distributed 1 215 AI kits in nine sub-regions of Uganda (Omara, 2023).

### Multiple ovulation and embryo transfer

The first successful cattle embryo transfers were reported by Umbaugh in 1949, and the first calf was born in 1951 (Umbaugh, 1949, Willett et al., 1951). In Africa, only seven countries (Côte d'Ivoire, Kenya, Madagascar, Rwanda, South Africa, Zambia, and Zimbabwe) report using this technology (Greyling et al., 2002, Rischkowsky and Pilling, 2007, MINAGRI, 2018). While farmers recognize embryo transfer's benefits for reducing generation intervals and enhancing genetic improvement, its adoption remains low due to economic infeasibility in predominantly smallholder production systems, despite the potential genetic and productivity gains.

#### Kenya

Embryo transfer in Kenya began in 1982 with imported embryos, but adoption was limited due to high costs and smallholder farming. In 1998, local donor cows were super-ovulated, and their embryos were transferred to local recipients (Kios, 2019). Larger-scale organized MOET programs started in 2005 and were led by the Agricultural Development Corporation. Between 2005 and 2008, Agricultural Development Corporation farms produced 138 transferable embryos with a 41% pregnancy rate (Kios, 2019). From 2007 to 2012, transferable embryos ranged from 0 to 11 per donor, with pregnancy rates peaking at 37% (Kios et al., 2013). In 2014, *in vitro* embryo production was introduced. Using ova from locally bred Boran cows, the protocol yielded a 46.5% conception rate and 17 healthy calves (Mutembei et al., 2015).

#### South Africa

Most data on ET in Africa, reported by the International Embryo Transfer Society, comes from South Africa, where ET is most common (Stroud and Bó, 2011). In 2009, 1 446 flushes produced 8 753 *in vivo* derived (**IVD**) and 100 *in vitro* produced (**IVP**) bovine embryos (Stroud and Bó, 2011). By 2018, IVD embryo transfers dropped to 6 931, while IVP embryos increased to 3 741 (Viana, 2019). Ankole cattle were introduced to South Africa in 2004 via embryo import from East Africa. This dual-purpose breed is prized for lean meat, quality milk, and resilience in harsh climates (Kriel, 2023).

#### Rwanda

A 2013 evaluation recommended using Ankole x Jersey crossbred cows as surrogates for embryo transfer in Rwanda (Kugonza et al., 2013). In 2017, 88 cows received imported Jersey embryos, with a 58% success rate (MINAGRI, 2018). From 2018 to 2019, 51 cows received embryos, resulting in 13 calves (26.6% success rate) (MINAGRI, 2019). In 2020/2021, 7 of 15 recipient cows successfully conceived (47% success), exceeding the 30% target, with 200 more embryos imported (MINAGRI, 2021). In 2021/2022, the success rate of embryo transfer in the country improved from 32–37.7% (MINAGRI, 2022).

### Genetic selection

A major challenge in cattle farming in Africa and developing countries is poor pedigree and performance record-keeping, which hinders animal performance evaluation (Rege, 2001). The feasibility of utilizing genomic selection in smallholder cross-bred cattle populations in Africa was examined and its potential use in the selection of young bulls was recommended (Brown et al., 2016). The applications of genomic testing in Africa are multiobjective and multidisciplinary including; the selection of donor females or sire bulls, evaluating the best cattle breed composition (grade) for different production systems and environments, genomic prediction for traits of interest, and identification of genomic signatures of adaptive introgression (Gorbach et al., 2010, Bahbahani et al., 2018, Mrode et al., 2021). The highlighted examples are the African countries that have utilized and reported the successful use of genomic selection in the improvement of cattle production.

#### Kenya

**Fig. 6:**
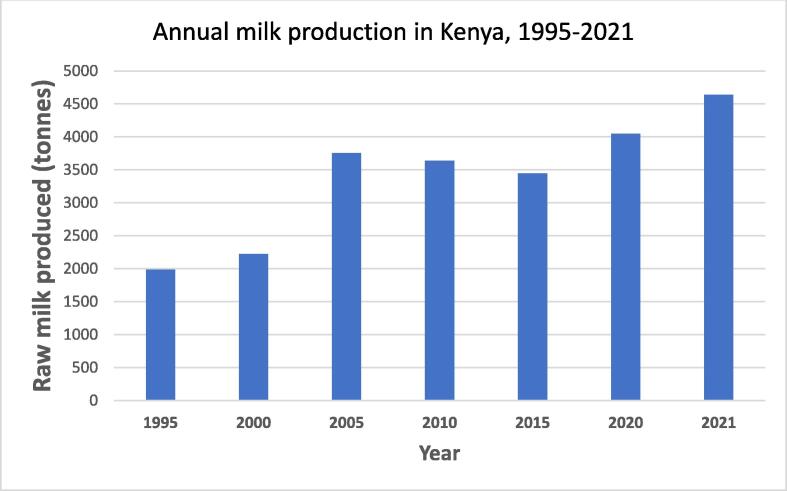
Trend of raw cattle milk production in Kenya. Data .

source: Faostat, 2023b

The dairy industry has grown significantly (Fig. 6), with over 4 million tons of milk produced in 2021 (Faostat, 2023b), 70% from smallholder farmers using cross-bred cows selected for traits like milk production, climate adaptation, and disease tolerance (Peeler and Omore, 1997, Omore et al., 1999). Pregenomic testing raised questions about the optimal breed composition for different environments. A 50 K SNP chip analysis of 195 animals (23 bulls, 172 cows, and calves) found large-scale farms had mostly Holstein cows, while small-scale farms had Holstein-Guernsey mixes, with little *Bos indicus* ancestry, raising concerns about adaptation to African climates and the sustainability of using these breeds for milk production in the long run (Gorbach et al., 2010).

The Dairy Genetics East Africa project, launched in 2011, evaluated cross-bred cows in Kenya. The joint collaborative effort with farmers combined performance data with breed composition genotypic data generated using the Illumina bovine high-density SNP assay to highlight the best breed options for specific production environments. The data identified low to medium-exotic breed mixes (< 50% exotic ancestry) as best for low and medium-production environments (Ojango et al., 2014). This highlighted the importance of long-term genetic improvement programs, leading to the African Dairy Genetic Gains (**ADGG**) initiative.

#### Tanzania

The ADGG project began in Tanzania in 2016 to create a sustainable national breeding program (ILRI, 2019). The project developed a digital system for collecting and analyzing phenotypic and genomic data from smallholder and medium-scale dairy farms (ILRI, 2019). By 2019, an estimated 15 690 farmers and 38 914 animals were registered, with monitoring of 13 589 farms and 26 433 animals (Mrode et al., 2020a). By 2021, registrations rose to 33 630 farmers and 66 917 animals, with 6 000 animals genotyped (Ojango et al., 2022). By 2023, over 260 000 test-day milk yields and 44 853 BW records were recorded from 79 269 animals (Msuta et al., 2023).

The ADGG platform used SNP assays for genotyping and genomic predictions (Brown et al., 2016, Ojango et al., 2019). Data from 5 268 genotyped cows and bulls indicated that cows with over 87% exotic genes produced twice the milk of those with less than 36% exotic genes. Calves born after 2014 averaged about 80% exotic genes, surpassing adult cows (Mrode et al., 2021). In 2020, data from 65 624 animals helped develop a selection index that was aimed at increasing milk production with reduced feed requirements (Mrode et al., 2020b). Currently, semen from top bulls is produced for artificial insemination of cows in smallholder farms. Overall, the ADGG program has led to a 54% increase in milk yield, from 6.7 to 10.34 L per cow per day (Msuta et al., 2023). A key benefit of ADGG has been enabling farmers to estimate the genetic merit of their cows through genomic predictions (Mrode et al., 2023).

#### Ethiopia

Ethiopia established its first national dairy cattle performance recording system in 2012, supported by the National Resources Institute, Finland. This program merged with the ADGG program in 2016, using ICT tools to capture phenotypic and genomic data in real-time (Gebreyohanes et al., 2021, Meseret et al., 2022). The ADGG program aimed to register at least 12 000 dairy herds. By 2019, it registered 12 576 farmers and 36 042 animals, monitoring 19 658 animals across 6 559 farms (Mrode et al., 2020a). By 2021, registrations increased to 71 215 farms and 105 924 animals, with 8 140 animals genotyped (Ojango et al., 2022). In 2022, the program registered 74 500 herds and 157 000 animals in 98 districts. Data from 440 000 test-day milk yields and 313 000 BW records were used to evaluate genomic estimated breeding values, ranking animals based on genetic superiority. These results were published in the national Cow and Bull Catalogue (Meseret et al., 2022). To promote superior genetics, three top-ranked bulls were included in the National Insemination Center for semen production, matching the quality of imported pure exotic AI bulls (Negussie et al., 2022). By 2022, 67 000 semen straws had been produced for breeding in 14 districts, with plans to expand further in the country (Meseret et al., 2022). The economic gain from utilization of the selected local superior bulls has been estimated at $644 200 and attributed to money saved through import substitution (Meseret et al., 2022). Since its inception, the genetic selection program has been successfully integrated and is still ongoing in smallholder and medium-scale farms in Ethiopia.

#### Senegal

To address high milk demand and low production, the Senegalese government launched a dairy improvement program to cross-breed indigenous cattle with high-yielding exotic breeds via artificial insemination. A major challenge was the lack of pedigree and performance data for selecting appropriate cross-breeds for various production systems. The Senegal Dairy Genetics project was established to identify optimal cattle breeds using a Bovine 50 K SNP assay. Results showed that smallholder farms (≤8 animals) with cross-bred indigenous zebu and *Bos taurus* breeds produced 7.5 times higher annual milk yields and 8 times greater household profits when managed well, compared to indigenous zebu under poor management (Marshall et al., 2016).

## Challenges in the integration and adoption of breeding technologies in Africa

The effective integration of cattle breeding technologies into dynamic African production systems is influenced by: (1) the type of production systems; (2) environmental conditions: (3) the economic capacity of the targeted population; (4) policies, guidelines, and regulatory frameworks available; (5) supply and distribution infrastructure; and 6) socio-cultural considerations. Several challenges faced during this integration in different African countries are highlighted below.

### Insufficient technical and personnel capacity

Breeding technologies like AI, MO, and ET require trained personnel for successful implementation. Low pregnancy rates often stem from varying skill levels of AI technicians (Allan et al., 2024). Many AI and ET programs in Africa have failed due to this issue (Madan, 2003, Tegegne et al., 2016, Allan et al., 2024). A review in 1989 highlighted a shortage of trained and motivated personnel as a barrier to integrating embryo transfer into breeding programs (Seidel and Seidel, 1989). In Rwanda, challenges in AI adoption included the capacities and motivation of inseminators, while Ethiopia faced technical capacity issues during its 2007 mass hormonal synchronization and insemination project (Tegegne et al., 2016).

Additionally, a 2019 survey found that 10% of respondents struggled to find inseminators, and over 24% experienced poor timeliness (MINAGRI, 2019). Madan (2003) noted a lack of trained technicians and researchers as a constraint in applying animal biotechnologies in Africa (Madan, 2003). In Botswana, a shortage of qualified ranch managers hampered AI use (Moreki and Ranko, 2011). Barriers in Kenya and Ethiopia included a shortage of AI technicians, technical failures by inseminators, and poor communication (Baltenweck et al., 2004, Baheriw et al., 2013, Ibrahim et al., 2014, Lijalem et al., 2015, Tesfaye et al., 2015). A recent study in Nakuru County, Kenya, identified inadequate knowledge and skills among inseminators regarding new breeding technologies as a gap in AI service utilization (Tellegen and Kimalit, 2023).

### Limited data on breed type, breed composition, and performance

Many smallholder farmers in Africa lack information on breed composition and suitable cross-breeds, hindering their ability to make informed germplasm choices (Seck et al., 2016). The absence of livestock databases, including breed types and compositions, has been a significant barrier to the success of new breeding technologies in developing countries (Seidel and Seidel, 1989). Furthermore, livestock improvement programs in Africa have struggled to meet their goals due to insufficient performance data and pedigree records (Muchadeyi et al., 2020). In Rwanda, 1.4% of farmers cited a lack of information about available breeds and bulls as a challenge contributing to low technology adoption (MINAGRI, 2019).

### Insufficient funding and resources

In a resource-limited world, high demand often outstrips supply, posing challenges for projects like cattle genetic improvement programs in Africa. Governments and funding partners prioritize urgent needs, resulting in less financial support for important but less urgent initiatives, such as livestock breeding. For instance, Senegal's National Artificial Insemination Program ceased after 2004 due to funding shortages, and only 11% of the necessary resources were mobilized for the Agricultural Leap for Food and Abundance project, which failed to meet its insemination targets (Seck et al., 2016). The livestock sector in Africa receives less than 10% of agricultural spending and under 1% of total government expenditure (AUIBAR, 2024). Africa's diverse production systems, ranging from smallholder to large commercial farms, often lack funding for new breeding technologies and resources like land (Seidel and Seidel, 1989). Small− and medium-scale farmers face difficulties securing personal financing and obtaining credit, as banks are hesitant to invest due to the complexities of these production systems.

### Low return on investment in biotechnology

Breeding technologies require farmers to make initial investments to realize economic benefits. In developing African countries, concerns about the costs of new breeding technologies and their returns discourage many farmers from adopting them, leading them to favor affordable, established methods. Smallholder farmers have found that the economic gains from integrating embryo transfer do not justify the required investments (Kahi and Rewe, 2008).To maximize profits, dairy farmers prefer female calves, but using unsexed semen only offers a 50% chance of achieving this. Farmers in Ethiopia and Uganda have linked the higher birth rates of male calves in their programs to artificial insemination, leading to decreased adoption of these services (Mugisha et al., 2014, Lijalem et al., 2015). Additionally, Ugandan farmers have noted that AI calves are often too fragile and too large for their dams to deliver without complications (Mbowa and Shinyekwa, 2012).

### Poor accessibility and availability of breeding technologies/services

The production and distribution of bovine semen in many countries are centralized in a few national centers. Farmers far from these centers rely on transported semen, facing challenges due to poor transport infrastructure (Kahi and Rewe, 2008). This limits timely access to semen and embryos, leading many to prefer natural mating despite the benefits of new breeding technologies. In Tanzania, farmers in the south depend on semen transported from the northern national insemination center, resulting in decreased AI usage (Mwanga et al., 2019). Similarly, in Uganda, reliance on the solitary center in Entebbe has diminished AI service use due to poor accessibility (OAGU, 2015). Poor maintenance of semen production and insemination centers further reduces service quality.

In Botswana, inadequate maintenance of AI facilities and long distances to AI centers contribute to the decline in AI service usage (Moreki and Ranko, 2011, Mocheregwa, 2016). In Ethiopia, the distance from AI stations and limited weekend services hinder adoption (Baheriw et al., 2013, Ibrahim et al., 2014, Lijalem et al., 2015, Tesfaye et al., 2015). In Kenya, the non-adoption of AI is linked to poor availability, high costs, and low conception rates from embryo transfers due to inadequate facilities and power outages (Baltenweck et al., 2004, Ongubo et al., 2015). Many farmers in Western Kenya (68%) prefer AI but choose natural mating for its accessibility and affordability (Lukuyu et al., 2019).

### Poor heat detection

Poor heat detection significantly impacts reproductive performance in Africa, leading to issues like repeat breeding, which hinders the adoption of AI technologies (Lijalem et al., 2015). Most smallholder dairy farmers rely on inefficient visual observations to detect estrus, with over 50% of Eastern African farmers using this method and fewer than 5% employing hormonal synchronization (Mwanga et al., 2019). Factors contributing to missed insemination opportunities include the absence of farmers in cow sheds, lack of knowledge about heat signs, late detection in the estrus cycle, and delayed arrival of inseminators (Mekonnin et al., 2015). In Ethiopia's Hydia zone, 28% of farmers cited poor heat detection as a barrier to using synchronization and AI services (Lijalem et al., 2015).

### Poor monitoring systems for breeding programs

Joint efforts involving public–private partnerships among African governments, international organizations, farmers, and stakeholders have led to the development of various cattle genetic improvement programs. However, despite initial successes, many programs have failed due to poor monitoring and follow-up, resulting in decreased adoption of effective breeding methods. This lack of follow-up also hampers data collection needed to assess the project's impacts, which can lead to reduced funding for similar initiatives and hinder scaling efforts. For instance, in Senegal, essential data on calving rates during the livestock support project were lost due to the absence of a birth monitoring system (Kouamo et al., 2009b).

### Natural disasters, civil and political conflicts

Natural disasters such as droughts and floods can and have influenced the adoption of biotechnologies globally. Breeding technologies targeting livestock can be derailed by high cattle mortalities attributed to poor nutrition, diseases, and physical trauma brought about by prolonged drought and floods. In Botswana, the low conception rate (61%) reported in 2017 was attributed to the severe famine that afflicted the country (Moreki et al., 2019). Madan (2003) identified political instability as one of the factors that may influence the limitation of biotechnology integration in developing countries. The civil conflict between Senegal and Mauritania in 1989 impeded the delivery of artificial insemination services to victims of the conflict and the displaced persons between 1996 and 1999 despite the funding provided for a livestock improvement project. During those 4 years, only 384 cows were inseminated under the project (Kouamo et al., 2009b).

### Underdeveloped legal frameworks, policies, and guidelines on biotechnology operations

The introduction of new biotechnologies requires clear guidelines and policies for importation, integration into breeding programs, and monitoring and evaluation systems to assess their impact. Regulatory frameworks should rigorously evaluate biotechnology products before approval, while logistical and management guidelines for imports and exports are essential. In many African countries, privatization of breeding technology importation has led to unclear monitoring systems, resulting in disorganized integration and failed breed improvement programs. For instance, a cross-breeding scheme in Mali, which involved local N’dama cows and improved breeds like Jersey and Red Steppe, failed due to chaotic importation of European breeds into a non-self-sustaining cattle population (Tamboura et al., 1982).

## Overcoming barriers to the adoption of cattle breeding technologies in Africa

### Capacity building of personnel in the African cattle production sector

Training a significant number of African animal health personnel and technicians in existing and new breeding technologies is crucial for cattle breeding across various production systems. Skilled personnel enhance the likelihood of attracting investment and support for cattle improvement programs, ensuring their sustainability after project funding ends. For example, the Vache du Faso program in Burkina Faso highlighted the importance of qualified technical staff for the effective implementation of breeding technologies such as artificial insemination (Allan et al., 2024).

### Introduction of cattle data programs

Farmer-integrated data platforms enable farmers to input animal data via mobile technology into a central server. These data help make informed breeding decisions, such as selecting genomic sires, choosing breeds for specific conditions, and optimizing production traits. The ADGG program in Kenya, Ethiopia, and Tanzania exemplifies this approach (Mrode et al., 2020b, Ojango et al., 2022, Msuta et al., 2023). Similar platforms could be developed by private companies distributing semen, with farmers trained to enter data and receive updates. To ensure sustainability, regular follow-ups, recruitment of new farmers, and collaboration between private companies, livestock ministries, and other stakeholders are essential for effective implementation and data sharing.

### Investment in cattle breeding technologies in Africa

National and local governments would need to allocate funding for the improvement of cattle production through cattle breeding programs, distribution, and adoption of new breeding technologies to farmers and research on improved breeding strategies. Public-private partnerships between government ministries and private companies, research institutes, and non-governmental organizations should be initiated to support national and regional cattle improvement programs.

### Affordable breeding technologies and ready markets in Africa

Before introducing new breeding technologies in African cattle production, several factors must be considered: the type of production system, farmers' priority needs, affordability of the technologies, and projected economic gains. These factors will guide decisions on appropriate breeding strategies, programs, and technologies for each region to ensure optimal adoption rates and return on investment. Additionally, the availability of downstream markets for cattle products like milk, meat, live animals, hides, and skins is crucial. Secured markets assure farmers of income and motivate higher acceptance and adoption of these breeding innovations.

### Secured supply and distribution of breeding technologies

Easy access to various breeding technologies will increase adoption rates among farmers. Establishing semen distribution centers at the community level ensures quick access to artificial insemination for smallholder and medium-scale farmers. This proximity reduces costs for technical personnel and farmers, increasing economic gains alongside genetic improvements in cattle. Regular maintenance and updates of the supply and distribution infrastructure are essential. This includes backup power and water supplies at distribution centers, all-weather roads, and well-maintained vehicles, equipment, and instruments for service delivery.

### Improved heat detection techniques

To improve heat detection in African cattle before breeding using sire bulls, artificial insemination, and embryo transfer, several strategies should be employed. Farmers can be trained in diligent record-keeping to anticipate potential heat days, which can be confirmed by skilled personnel. Training should also cover factors influencing cow cycling, such as management practices, to improve heat detection. Estrus synchronization is an effective method for medium- and large-scale production systems or programs involving many smallholder farmers, allowing for fixed-time AI without heat detection.

### Monitoring and evaluation of breeding programs

Evaluating the results of breeding programs involves assessing their production, social, and economic impacts on individual, local, and national levels. These evaluations provide insights for amending strategies, objectives, and activities to address gaps and meet producers' demands. Ongoing monitoring ensures project goals are being met and stakeholder participation remains active. This process helps identify bottlenecks and challenges, allowing for strategies to address them. Mid- and end-of-program evaluations inform decisions on program continuation, funding allocations, and potential changes to the breeding programs.

### Development of disaster response action plans

Livestock ministries in Africa should develop disaster response action plans for various situations, including natural disasters, disease outbreaks, and civil conflicts. These plans should outline response protocols for animals in different production systems and breeding programs. They should include details on funding for activities like vaccination, treatment, cattle movement, and farmer reimbursements to recover animals or start new breeding programs. These efforts should be supported collaboratively by stakeholders, including institutions, private companies, and non-governmental organizations.

### Development of legal frameworks, policies, and guidelines on biotechnological operations

African countries need to develop and update policies and guidelines for introducing, implementing, and utilizing new breeding technologies. National and local governments should lead these efforts, supported by stakeholders such as private institutions and organizations. These policies and guidelines will outline requirements for integrating breeding technologies into existing and new programs, preventing technological overlap in specific regions, and facilitating efficient monitoring and evaluation. This ensures optimal benefits for farmers and other stakeholders in the cattle production supply and value chains. New policies can be adapted from countries with similar cattle production systems.

## Conclusions

In conclusion, integrating various cattle breeding strategies such as cross-breeding, artificial insemination, embryo transfer, and genetic selection has significantly improved genetic diversity and productivity in dairy systems across 12 Sub-Saharan African countries. However, the case studies presented may not fully represent the current situation, and many successful breeding programs lack recorded data, potentially skewing perceptions of technology adoption. While challenges like limited funding, technical skills, and economic feasibility hinder the broader adoption of some technologies, the combined use of existing breeding methods has produced measurable productivity and economic benefits. Successful examples in Africa show these strategies can be adapted to other tropical dairy systems, while failures provide lessons for future initiatives. Addressing gaps in funding, collaboration, and program follow-up presents opportunities to engage stakeholders, refine breeding programs, and enhance dairy production. Ultimately, the positive impact of breeding technologies on dairy and economic development highlights the potential for increased investment and research, supported by stronger public–private partnerships to advance Africa's dairy industry.

## Ethics approval

The authors did not use any live subjects to conduct this review.

## Data and model availability statement

None of the data was deposited in an official repository. The data that support the study findings are publicly available. Information can be made available from the authors upon request.

## Declaration of Generative AI and AI-assisted technologies in the writing process

During the preparation of this work the author(s) did not use any AI and AI-assisted technologies.

## Author ORCDIDs

**Emily Kathambi:**https://orcid.org/0000-0002-2086-7344.

**Tad Sonstegard:**https://orcid.org/0000-0002-6446-9276.

**Peter Larsen:**https://orcid.org/0000-0002-3634-3625.

## CRediT authorship contribution statement

**E.K. Kathambi:** Writing – review & editing, Writing – original draft, Conceptualization. **T.S. Sonstegard:** Writing – review & editing, Funding acquisition. **P.A. Larsen:** Writing – review & editing, Supervision, Funding acquisition, Conceptualization.

## Declaration of interest

EKK and PAL declare no conflict of interest. TSS was employed by Acceligen™ when this study was carried out.
